# Astrocyte–Oligodendrocyte–Microglia Crosstalk in Astrocytopathies

**DOI:** 10.3389/fncel.2020.608073

**Published:** 2020-11-19

**Authors:** Dieuwke Maria de Waard, Marianna Bugiani

**Affiliations:** Department of Pathology, VU Medical center, Amsterdam UMC, Amsterdam, Netherlands

**Keywords:** Aicardi–Goutières syndrome, Alexander disease, astrocytopathies, cellular crosstalk, megalencephalic leukoencephalopathy with subcortical cysts, oculodentodigital dysplasia, vanishing white matter

## Abstract

Defective astrocyte function due to a genetic mutation can have major consequences for microglia and oligodendrocyte physiology, which in turn affects the white matter integrity of the brain. This review addresses the current knowledge on shared and unique pathophysiological mechanisms of astrocytopathies, including vanishing white matter, Alexander disease, megalencephalic leukoencephalopathy with subcortical cysts, Aicardi–Goutières syndrome, and oculodentodigital dysplasia. The mechanisms of disease include protein accumulation, unbalanced secretion of extracellular matrix proteins, pro- and anti-inflammatory molecules, cytokines and chemokines by astrocytes, as well as an altered gap junctional network and a changed ionic and nutrient homeostasis. Interestingly, the extent to which astrogliosis and microgliosis are present in these astrocytopathies is highly variable. An improved understanding of astrocyte–microglia–oligodendrocyte crosstalk might ultimately lead to the identification of druggable targets for these, currently untreatable, severe conditions.

## Introduction

**FIGURE 1:**
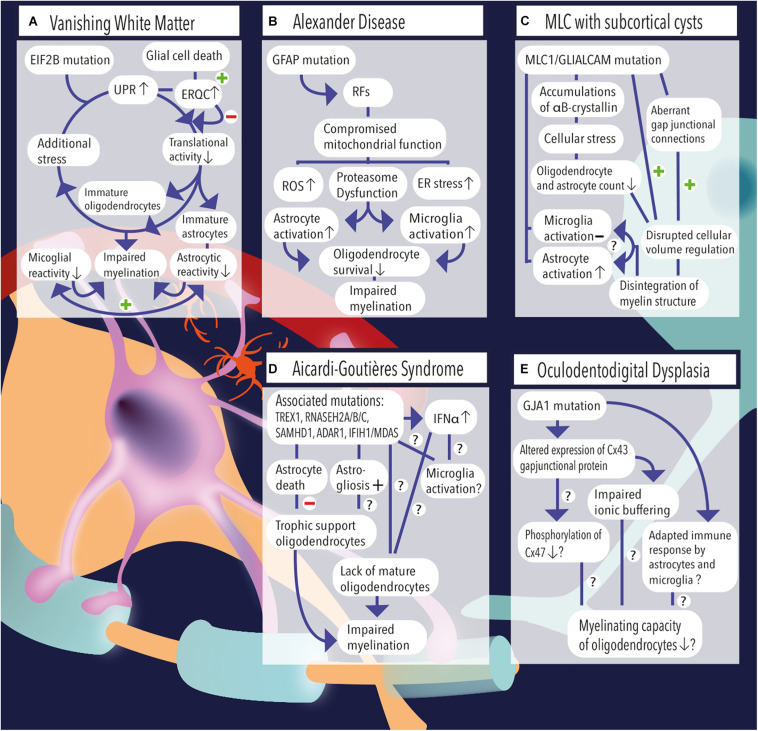
**(A)** Vanishing white matter is caused by recessive mutations in the *EIF2B* gene. Induction of the unfolded protein response (UPR) causes lowering of the cellular translational activity and results in glial cell immaturity (e.g., astrocytes and oligodendrocytes). Additional stress (febrile infections or minor head trauma) further induces the cycle of cellular stress. Rescue mechanisms such as endoplasmic reticulum quality control (ERQC) are activated to escape a vicious UPR cycle and promote glial cell death if cellular damage exceeds the threshold for recovery. Vanishing white matter specimens characteristically show reduced astrocytic and microglial reactivity. Together, the lack of mature astrocytes and oligodendrocytes as well as the absence glial cell activation impairs the process of myelination. **(B)** Overexpression of GFAP due to homozygous mutations in the *GFAP* gene causes the formation of Rosenthal fibers (RFs) in the cytosol of astrocytes. Astrocytic pathology leads to an impaired respiratory chain complex (i.e., impaired mitochondrial function), which is indicated by increased levels of reactive oxygen species (ROS), endoplasmic reticulum (ER) stress, and proteasome dysfunction as pointed out by activation of kinase pathways. Pathological hallmarks of Alexander disease are prominent activation of astrocytes and microglia. It is hypothesized that sustained activation of both cell types has a detrimental effect on oligodendrocyte survival and contribute to a lack or loss of myelin. **(C)** *MLC1* or *GLIALCAM* mutations are causally related to the development of megalencephalic leukoencephalopathy (MLC) with subcortical cysts. Accumulations of αB-crystallin have been observed in the cytosol of MLC astrocytes. The resulting cellular stress might relate to the observed reduction in the number (#) of oligodendrocytes and astrocytes. Although microglial activation is absent, MLC specimens demonstrate substantial astrogliosis, which usually are physiologically coupled events. Dysregulation of fluid excesses after neuronal depolarizations (i.e., disrupted cellular volume regulation) results in disintegration of the myelin layers and presence of intramyelinic vacuoles. **(D)** Aicardi–Goutières syndrome’ associated mutations include *TREX1*, *RNASEH2A*, *RNASEH2B*, *RNASEH2C*, *SAMHD1*, *ADAR1*, and *IFIH1/MDAS*, which result in an increased and dysregulated production of interferon-alpha (IFNα), which might be toxic to oligodendrocytes and might induce sustained activation of microglia. Furthermore, AGS mutations have been related to increased astrogliosis and astrocyte death. As oligodendrocytes are highly dependent on trophic support by neighboring astrocytes, their survival rate is severely affected and myelinating capacity is limited. Yet, it is unclear whether Aicardi–Goutières-associated mutations might (also) have an effect on microglial activation or oligodendrocyte maturation directly. **(E)** A mutation in the *GJA1* gene, which is associated with oculodentodigital dysplasia, causes altered expression of the gap junctional hemichannel Cx43, which is abundantly expressed in astrocyte–astrocyte and astrocyte–oligodendrocyte contacts. A direct consequence of altered Cx43 expression might be a phosphorylation defect of Cx47 on the oligodendrocyte’ membrane, which in turn negatively affects their myelinating capacity. In addition, impaired ionic buffering of K^+^ and Na^+^ ions might affect oligodendrocyte homeostasis and affect the process of myelination. In addition, adaptations in the immunological response of astrocytes and microglia have been observed and related to altered expression of Cx43.

The brain’s white matter is composed of myelinated axons, glial cells, and blood vessels embedded in the extracellular matrix. All glial lineage cell types, i.e., astrocytes, oligodendrocyte precursor cells (OPCs), mature oligodendrocytes, microglia, NG2-glia, and ependymal cells, play an important role in health and disease. A variety of regulatory functions related to brain homeostasis have been attributed to healthy astrocytes, the most abundant glial cell type in the human brain. Briefly, astrocytes facilitate repetitive firing of neurons by buffering extracellular excesses of potassium (K^+^) and glutamate (Bellot-Saez et al., 2017). Astrocytes maintain the tissue pH at a steady level and have an important metabolic function, illustrated by their share in glycogen storage (Wang and Bordey, 2008) and production of sterols and lipids (Dietschy and Turley, 2004). In addition, they regulate the influx of soluble molecules from the cerebrospinal fluid to the brain tissue and control cerebral blood flow dynamics (Howarth, 2014). To this end, astrocytes maintain an intimate molecular conversation with other glial cell types. The astrocyte–oligodendrocyte interaction is important for the oligodendrocytes to apply their major task: the production of the myelin sheath of axons. Microglia are highly dependent on signaling molecules secreted by astrocytes to carry out their immunological function. Interactions between cells of glial lineage are complex and multidirectional in nature. Aberrant exchange of signaling molecules, as a result of a primary defect in the astrocytic genome, can result in severe neurological disease. The current review explores the pathophysiological consequences of DNA mutations primarily affecting astrocyte function, also referred to as astrocytopathies (van der Knaap and Bugiani, 2017). The astrocytopathies highlighted in this review include vanishing white matter (VWM), Alexander disease (AxD), megalencephalic leukoencephalopathy with subcortical cysts (MLC), Clc-2-related disease, Aicardi–Goutières syndrome (AGS), and oculodentodigital dysplasia (ODDD) (Figure 1). Every section starts with a neuropathological description of one of the aforementioned astrocytopathies and subsequently discusses the mediators of (bidirectional) cellular communication between astrocytes and oligodendrocytes, astrocytes and microglia, and oligodendrocytes and microglia, in relation to disease progression.

## Neuropathology of Vanishing White Matter

Vanishing white matter is a leukodystrophy that affects young children and manifests with progressive neurological deterioration with cerebellar ataxia, spasticity, and cognitive decline. The clinical course typically worsens upon stressful events, such as febrile infections and minor head trauma. The neuropathology of VWM involves myelin thinning and lack of myelin, myelin vacuolation (i.e., intramyelinic edema), and tissue rarefaction and cavitation. The cerebral cortex is spared, but subcortical gray matter structures (e.g., the thalamus, midbrain, and pons) may be affected. In lesioned areas, oligodendrocytes are reduced in number, although they are profoundly present in subcortical U-fibers (i.e., connections between adjacent gyri) and relatively spared white matter areas (e.g., optic system, anterior commissure, corpus callosum, and internal capsules). In contrast, the number of OPCs is markedly increased around areas of cavitation. Astrocytes are also reduced in number and show a dysmorphic phenotype with broad blunt processes. Astrocytes in VWM brain specimens characteristically express glial fibrillary acidic protein (GFAP)δ, vimentin and nestin, but do not overexpress GFAPα, and S100β, indicating an immature phenotype. Furthermore, expression of αB-crystallin is observed in astrocytes. Reactive astrogliosis and microglia cell activation are not typically observed in VWM and only mildly in the gray matter (Bugiani et al., 2010, 2011). Recessive mutations in one of the five genes encoding the subunits of eukaryotic translation initiation factor 2B (*EIF2B* subunit α-γ) are causally related to the disease (van der Knaap et al., 2002). The eIF2B protein is essential for the translation of messenger RNA (mRNA) into protein under normal and stress conditions. Under control of the eIF2B enzyme, guanosine diphosphate (GDP)-bound eIF2 is exchanged to guanosine triphosphate (GTP), and the binding of initiator methionyl-transfer-RNA (Met-tRNAi) to the small ribosomal subunit is facilitated. This construct is required to start protein synthesis. Apart from the crucial role of eIF2B in protein translation initiation, eIF2B is involved in the unfolded protein response (UPR), a cellular pathway that is activated upon accumulation of inaccurately folded proteins in the endoplasmic reticulum (Schröder and Kaufman, 2005). *EIF2B* mutations result in lowering of translational activity and elevated activation of the UPR. The astrocytic UPR consists of three signaling routes. Initially, only the PERK-eIF2a-ATF4-CHOP UPR pathway was shown to be involved in the pathophysiology of VWM (van der Voorn et al., 2005). Follow-up research revealed that three UPR pathways are activated in the VWM brain (van Kollenburg et al., 2006). van Kollenburg et al. (2006) demonstrated involvement of PERK, IRE1, and ATF6 signaling cascades in distinct brain autopsy samples from VWM patients. Using immunohistochemistry, the UPR was localized predominantly in oligodendrocytes and, to a lesser extent, astrocytes (van Kollenburg et al., 2006). Consistent with the neuropathological observation, it was found that elevated activation of the glial UPR contributes to abnormal proliferation and apoptosis of oligodendrocytes in VWM (Van Haren et al., 2004). In astrocytes, lowered translational activity might directly affect the formation of cytoskeletal proteins. Healthy astrocytes express, among others, GFAP as a major protein of their cytoskeleton. In VWM, mutated *EIF2B* compromised GFAP-pan expression in astrocytes (Dietrich et al., 2005). Dietrich et al. (2005) demonstrated that, using RNAi, disrupted eIF2B5 function severely affected the generation of GFAP-pan in human glial progenitor cells. In line with this, Bugiani et al. (2011) observed profound expression of GFAPδ protein in VWM astrocytes as well as elevated GFAPδ transcripts. Together, there is evidence for alternative splicing of GFAP in VWM astrocytes, resulting in an elevated GFAPδ/GFAPα ratio. The presence of GFAPδ is associated with interfilament network instability (Perng et al., 2008) and, in case of collapse, altered cell morphology but not cell proliferation and migration (Moeton et al., 2016). In addition to cytoskeleton defects, defective mitochondrial function, as pointed out by a decrease in oxidative phosphorylation, has also found to be present in VWM astrocytes and oligodendrocytes (Raini et al., 2017; Herrero et al., 2019). Oligodendrocytes and astrocytes are highly dependent on adequate supply of energy resources, as their major tasks are the production of myelin proteins and transcription, translation, and secretion of growth factors, cytokines, and chemokines, respectively (Elroy-Stein, 2017). During the process of maturation to oligodendrocytes, oxidative phosphorylation is increased in OPCs (Schoenfeld et al., 2010; Rinholm et al., 2011). Therefore, compromised mitochondrial functionality can be related to the disproportional number of OPCs compared to mature oligodendrocytes in patients’ brain tissue (Bugiani et al., 2010, 2011). This argues for a wide view on the effects of an *EIF2B* mutation in astrocytes and oligodendrocytes, in any case including defects in the cytoskeleton and energy deficits due to a compromised mitochondrial respiratory chain complex.

### Astrocyte–Oligodendrocyte Crosstalk in Vanishing White Matter

As the primary manifestation of VWM is the lack of myelin, the relationship between astrocytes and oligodendrocytes is increasingly being investigated. A study of Dooves et al. (2016) confirmed a primary role of astrocytes in the pathophysiology of VWM by assessing the intrinsic and extrinsic cues on OPC maturation in astrocyte–oligodendrocyte cocultures, obtained from VWM mice. *In vivo* data pointed out the presence of immature astrocytes before other histological hallmarks of disease and hereby confirmed that OPC dysmaturation is a result of astrocytic pathology. *In vitro*, the combination of VWM astrocytes and wild-type OPCs resulted in significantly lowered expression of myelin basic protein (MBP) and myelin oligodendrocyte glycoprotein (MOG), indicating an oligodendrocyte maturation defect. On the contrary, VWM OPCs were able to differentiate in the presence of wild-type astrocytes, indicating that the OPC maturation defect is not intrinsic. The authors stated that factors secreted in the culture medium by VWM astrocytes contributed to the observed halt in OPC maturation. Although many factors from astrocytic origin influence OPC survival, oligodendrocyte differentiation, maturation, and myelination (reviewed by Harlow and Macklin, 2014), this study highlighted a role for hyaluronan in OPC maturation. Consistently, Bugiani et al. (2013) observed accumulation of high molecular weight hyaluronan and an increased OPC/oligodendrocyte ratio in lesioned areas of the VWM brain (Bugiani et al., 2013). Additional evidence for the role of brain extracellular matrix molecules (ECM) in VWM pathology comes from Moeton et al. (2016). They showed that GFAPδ expression was associated with a decrease in plectin and an increase in laminin (Moeton et al., 2016). A causal relationship between OPC maturation and ECM molecules was demonstrated by Cho et al. (2019) using a state-of-the-art cell culture model. Oligodendrocytes were generated from induced pluripotent stem cells (iPSC) in a matrix of decellularized human brain tissue, of which the latter contained various types of collagen, glycoproteins, and proteoglycans. These oligodendrocytes were capable of producing a functional myelin sheath around induced neurons in cocultures (Cho et al., 2019). Thus, ECM proteins play a pivotal role in the communication between astrocytes and oligodendrocytes and influence the pathogenesis of VWM. Of note, recently, an advanced *in vitro* model for VWM was established using patient-derived iPSCs (Zhou et al., 2019), and secretomics is considered to rise in importance in this field of research (Song et al., 2019). Combining these strategies might lead to an ameliorated *in vitro* model for VWM and has the possibility to improve our understanding of astrocyte–ECM–oligodendrocyte crosstalk in this astrocytopathy. Another aspect of astrocyte–oligodendrocyte communication involves the secretion of inflammatory mediators. Normally, interleukin (IL)-6 and IL-1β are proinflammatory cytokines that are produced by activated glial cells upon injury and stimulate the transition of OPCs to mature oligodendrocytes (Williams et al., 2007). IL-1β stimulates OPCs to differentiate into myelinating oligodendrocytes via the induction of ciliary neurotrophic factor (CNTF) and insulin-like growth factor (IGF-1) (Louis et al., 1993). Moreover, IL-1β regulates the synthesis of myelin by mature oligodendrocytes and promotes their survival (Mason et al., 2001). IL-6 is an evenly important cytokine for oligodendrocyte survival and has a neuroprotective role (Barres et al., 1993; Chen and Trapp, 2016). Interestingly, pathological reports on VWM pointed out the absence of astrogliosis and microglial activation (Bugiani et al., 2010). To date, it is not fully understood how the absence of astrocyte and microglia reactivity in VWM affect the myelinating capacity of oligodendrocytes, but it is known that *EIF2B*-mutated glia demonstrate a compromised inflammatory response (Cabilly et al., 2012). Cabilly et al. (2012) investigated the effect of lipopolysaccharide (LPS) intraperitoneal injection (mimicking a febrile infection) on glial reactivity in mice brain harboring an *EIF2B* mutation. Using primary glial cell cultures, it was found that the secretion of inflammatory mediators after LPS administration was significantly reduced compared to controls. Specifically, upon exposure to LPS, mRNA transcripts of IL-6 and tumor necrosis factor (TNF)α were upregulated at a similar rate in mutated astrocytes compared to wild-type controls. However, intracellular levels of IL-6 were significantly lowered in mutated astrocytes. Using ELISA, it was observed that the secretion of IL-6 and TNFα in the culture medium was diminished in astrocyte cell cultures obtained from mice with mutant *EIF2B5*. Similar results were obtained for microglia. *EIF2B*-mutated microglia did not reach their characteristic state of activation following LPS administration. This was illustrated by reduction in intracellular IL-6 and IL-1β in mutant compared to wild-type microglia. Accordingly, the levels of secreted IL-6 and TNFα in the culture medium of mutated microglia were significantly lowered compared to control. These findings strongly suggest that EIF2B mutations result in poor astroglia activation and an impaired capability of astrocytes and microglia to secrete cytokines upon injury. As stated before, these cytokines support the differentiation of OPCs and survival of mature oligodendrocytes and induce myelination. Thus, their absence in VWM might exaggerate oligodendrocyte dysfunction. Overall, the exact relationship between astrocytes and OPCs or oligodendrocytes has not been fully elucidated, but adaptations to the ECM and reduced secretion of inflammatory signaling molecules due to astrocyte dysfunction have been proposed to play an essential role in aberrant astrocyte–oligodendrocyte communication in VWM.

### Astrocyte–Microglia Crosstalk in Vanishing White Matter

Microglia are the sensors of subtle changes in the brain microenvironment. Upon activation, microglia act as cleansers of pathogens, damaged cells, and cellular debris. To execute this task properly, microglia maintain an intimate molecular conversation with astrocytes and vice versa (Peferoen et al., 2014; Jha et al., 2019). Cytokines and chemokines are produced and secreted by both glial cell types and bidirectionally influence cellular function (Zhang et al., 2010). Their relationship is best illustrated by the regulatory role of microglia in the degree of reactive astrocytosis upon injury. It is assumed that microglia activation precedes and promotes astrocyte reactivity. In turn, activated astrocytes facilitate activation of microglia (Liu et al., 2011). However, in the VWM brain, there is meager astro- and microgliosis. The partnership of astrocytes and microglia in the formation of a glial scar upon injury is hampered or not even present. Next to the secretion of cytokines, innate immune pattern recognition receptors (PRRs) are important in glial communication. PRRs recognize molecules on the cell membrane of pathogens and activate cellular crosstalk between microglia and neighboring astrocytes to promote their elimination (Kigerl et al., 2014). An example of a PRR family expressed by microglia and astrocytes are the Toll-like-receptors (TLRs) (Farina et al., 2007; Fiebich et al., 2018). It was found that hyaluronic acid, which is abundantly expressed in VWM lesions, is a ligand for TLR2 and TLR4 (Termeer et al., 2002; Scheibner et al., 2006). In VWM, an excess of hyaluronan might inhibit TLR4-signaling in glia, thus limiting the production of cytokines and chemokines upon simulation by LPS (Austin et al., 2012; Cabilly et al., 2012). As it is still unknown whether the absence of proinflammatory mediators secreted by microglia progress or reduce the tissue injury in VWM, an avenue for future research might be to map the secretome of *EIF2B*-mutated microglia, to assess the effect of individual molecules on VWM astrocytes, and to test the modulating effect of ECM molecules in this relationship. Astrocyte–microglia crosstalk also plays an important role in axon myelination by regulation of oligodendrocyte homeostasis (Domingues et al., 2016). Activated astrocytes attract microglia to sites of injury via secretion of chemokines CCL2 and CXCL10 (Tanuma et al., 2006). Upon reaching the location of tissue injury, microglia are able to phagocyte myelin. The absence of microglial activation might therefore negatively influence oligodendrocyte homeostasis. Further discussion on microglia–oligodendrocyte crosstalk is described in the following section.

### Microglia–Oligodendrocyte Crosstalk in Vanishing White Matter

In VWM, microglial reactivity is reduced to a minimum. Normally, upon activation, microglia secrete factors that influence oligodendrocyte physiology in multiple ways. The signaling molecules include proinflammatory mediators, such as glutamate, matrix metalloproteinases, reactive oxygen and nitrogen species, excitotoxins, chemokines, and cytokines (Nayak et al., 2014). The primary goal of these molecules is initiating a defense against invading pathogens; however, they also influence the function of adjacent glia (Peferoen et al., 2014; Jha et al., 2019). An example of microglia–oligodendrocyte crosstalk was shown in a study of Pang et al. (2010), where LPS-induced microglial activation was associated with arrested OPC proliferation and increased OPC death (Pang et al., 2010). Additionally, studies in mice have shown that the absence of microglia negatively affect white matter repair after injury, indicating the dependence of oligodendrocytes on microglia in remyelination processes (Tanaka et al., 2013; Li et al., 2005). In contrast, neuropathological studies on VWM reported meager microglial activation and an increase in the number of OPCs (Bugiani et al., 2010, 2011). A failure to reach microglial activation might contribute to the observed increase in OPCs. The lack of microgliosis in VWM results in reduced microglial secretion of TNFα and IL-1β (Cabilly et al., 2012). The corresponding receptors, TNF-R1 and IL-1R1, are expressed on oligodendrocytes. Yet, the consequences of the diminished presence of the ligands TNFα and IL-1β on oligodendrocyte dysfunction in VWM have not been elucidated. Besides the notion that microglia show a ramified, non-activated morphology in VWM, little is known about the expression of receptors on the cell membrane of microglia in VWM. As a consequence of demyelination or cell death, oligodendrocytes may release nucleotides that are recognized by ionotropic P2X and metabotropic P2Y purigenic receptors expressed on microglia (Monif et al., 2010). P2-type receptors are essential drivers of microgliosis and chemotaxis of microglia to sites of injury (Nayak et al., 2014; Chun et al., 2018). The expression of these receptors on microglia in VWM tissue have not been studied in detail; therefore, the knowledge about their share in microglia–oligodendrocyte crosstalk in this disease is still inconclusive.

## Neuropathology of Alexander Disease

A mutation in the GFAP protein coding region can cause a major accumulation of this essential interfilamentous protein and, as a consequence, primary dysfunction of astrocytes (Brenner et al., 2001). The here referenced pathology was named Alexander disease (AxD), after W. Steward Alexander, and is characterized by a lack or loss of myelin and the presence of Rosenthal fibers (RFs) within the cytosol of astrocytes (Alexander, 1949). Clinically, two subtypes of AxD have been recognized. One with infantile onset (type I), which presents with macrocephaly, seizures, and motor as well as cognitive decline, and a late-childhood or adult onset form (type II), which is associated with bulbar and cerebellar dysfunction and delayed development of cognitive problems. In terms of neuropathology, types I and II can be distinguished based on the degree of white matter injury. In type I, severely white matter abnormalities have been described, mainly affecting the long range white matter tracts in both hemispheres, although cerebellar white matter and the spinal cord may be involved, too. Astrocytes are typically enlarged and commonly show multiple nuclei. Type II is best described as a degenerating white matter disease with a relatively mild course. Here, tissue degeneration is concentrated in the periventricular regions, brainstem and cerebellum. In both subtypes, high amounts of RFs have been observed within astrocyte cell bodies, in the cerebral hemispheric and deep cerebellar white matter (Sosunov et al., 2018). The major components of RFs in AxD are mutated GFAP (i.e., R239C or R416W GFAP), vimentin, αB-crystallin, and hsp27 (small heat shock proteins) (Der Perng et al., 2006; Heaven et al., 2016; Sosunov et al., 2017). Normally, αB-crystallin and hsp27 are chaperone proteins that prevent filament aggregation (Koyama and Goldman, 1999); however, in the presence of the GFAP-mutated isoforms, αB-crystallin and hsp27 become part of the aggregates themselves (Der Perng et al., 2006). Hsp27 is an important protein in the preservation of mitochondrial function (Paul et al., 2002) and regulation of proteasomal activity (Parcellier et al., 2003). Therefore, it was hypothesized that RFs compromise mitochondrial function in AxD and relate to increased levels of cellular oxidative stress (Sosunov et al., 2017). Supporting evidence included the observation of activated mitogen-activated protein kinase (MAPK), P38, and c-JUN N-terminal kinase (JNK) pathways in AxD and their association with inhibition of the proteasome function (Tang et al., 2006, 2010). Proteasome dysfunction causes endoplasmic reticulum stress, and this might induce the expression of kinase pathways in AxD again (Menéndez-Benito et al., 2005; Sekine et al., 2006; Sosunov et al., 2018). Furthermore, elevated levels of oxidative stress in AxD astrocytes derived from animal models have been described (Wang et al., 2015). Unfortunately, specific data on mitochondrial function in *in vivo* models of AxD are not yet available. Overall, proteasome dysfunction, endoplasmic reticulum stress, and oxidative stress all contribute to glial cell activation (Jansen et al., 2014; Sprenkle et al., 2017). A study by Hagemann et al. (2005) demonstrated enhanced expression of inflammation- and cellular-stress-related genes in transgenic mice that overexpressed GFAP and presented with RFs (Hagemann et al., 2005). Olabarria et al. (2015) examined the inflammatory response of astrocytes and microglia in a double-mutant AxD mouse model (GFAP^Tg^; GFAP^±R236H^) using total tissue RNA-seq. In short, of the top 100 increased transcripts, 32% was annotated as related to cytokines, chemokines, and chemotaxis, pointing out an increased immune response in AxD. The cell-specific transcript analysis revealed a marked increase in *CD44*, *Vimentin*, *GFAP*, *ALDH111*, and *GLAST* in AxD astrocytes, whereas a decrease in gene transcripts coding for aquaporin 4, glutamate transporter-1 (GLT-1), connexin (Cx)43, Cx30, and glutamine synthetase was found. For AxD microglia, transcripts of genes related to Iba1, CD11, C3R, CD18, CD200, P2Y6, CD68, CX3CR1, and P2Y12 expression were elevated compared to WT microglia (Olabarria et al., 2015). These studies are in line with the observation of activated astrocytes and microglia in neuropathological reports on AxD (Sosunov et al., 2018). In summary, the formation of RFs, activation of kinase pathways and subsequent proteasome dysfunction, increased endoplasmic reticulum stress, and subsequent production of reactive oxygen species contribute to elevated levels of cellular stress in AxD and compromise astrocyte function. The consequences of this primary astrocyte dysfunction on oligodendrocyte and microglial physiology is further described below.

### Astrocyte–Oligodendrocyte Crosstalk in Alexander Disease

The astrocytes’ ability to maintain physiological homeostasis is important for oligodendrocyte survival (Nutma et al., 2020). In a double mutant mouse model of AxD, caspase 3-immunopositive oligodendrocytes were detected, which is indicative of oligodendrocyte apoptosis (Sosunov et al., 2018). Oligodendrocyte survival is highly dependent on astrocyte buffering of glutamate and potassium from the extracellular milieu. Glutamate that is released by axons after neuronal excitation directly affects OPC and oligodendrocyte physiology via methyl-4-isoxazolepropionic acid (AMPA) and *N*-methyl-D-aspartate (NMDA) receptors (Fannon et al., 2015; Gautier et al., 2015). In addition, a rise in the levels of extracellular K^+^, which is a secondary to elevated glutamate levels, can affect oligodendrocyte function. Here, the right balance is of utmost importance, and an excess of glutamate or K^+^ might have detrimental effects. AxD astrocytes express less GLT-1/excitatory amino acid transporter 2 (EAAT2) glutamate transporters on their cell membrane (Tian et al., 2010; Sosunov et al., 2013) and are therefore less capable of adequate uptake of glutamate and potassium (Tian et al., 2010). As a result, oligodendrocytes will try to take up excessive K^+^, although this comes at the cost of cytosolic acidification by H^+^ and an increased chance of cellular death. Thus, a diminished ability of astrocytes to handle glutamate and potassium excesses can lead to oligodendrocyte death in AxD.

As mentioned earlier, astrocyte–oligodendrocyte crosstalk is evenly important for OPC transition to mature oligodendrocytes, and this communication is influenced by ECM molecules. Similar to VWM pathology, in AxD mouse models, deposition of glycosaminoglycan hyaluronan have been observed, and the CD44 receptor was present in short-branched AxD astrocytes (Sosunov et al., 2018). In VWM, accumulation of hyaluronan was associated with a halt in OPC maturation and a lack of functional oligodendrocytes. Based on the similarity in pathophysiological mechanism, in AxD, OPC maturation might also be inhibited due to accumulations of the glycosaminoglycan hyaluronan. Finally, astrocytic support of oligodendrocytes is essential for the process of myelination. However, because AxD murine models do not represent the myelination defects observed in patients (Messing et al., 2012), little is known about the aberrant communication of astrocytes and OPC or oligodendrocyte function. Using iPSC-derived astrocytes, Li L. et al. (2018) recapitulated AxD pathology characteristics *in vitro* and found that AxD astrocytes inhibit OPC transition into mature oligodendrocytes and myelination. The study attractively showed a reduced number of O4^+^ OPCs (a marker for the transition of OPCs to oligodendrocytes) in cocultures of iPSC AxD astrocytes with OPCs compared to control. The authors eliminated OPC apoptosis as a reason and pointed out diminished proliferation rates as the causal factor for the detected difference in OPC maturation. Furthermore, the transcriptome of AxD astrocytes was compared to control astrocytes and revealed a set of 232 differentially expressed genes, of which a subgroup was validated using real-time quantitative PCR (RT-qPCR). Next, the astrocyte-secreted protein CHI3L1 was revealed to play a modulating role on OPC maturation and myelination, and its increased expression was validated in an AxD patient brain tissue. Interestingly, blocking CHI3L1 activity from the culture medium using a neutralizing antibody reversed the inhibitory effect on OPC maturation. Moreover, knockdown of CHI3L1 in AxD astrocytes using short hairpin RNAs resulted in enhanced proliferation of OPCs, a higher number of mature oligodendrocytes, and an improved myelinating capacity. In addition, blocking of the corresponding receptor, CRTH2, on OPCs rescued the pathological phenotype (Li L. et al., 2018). To conclude, these studies illustrated the fundamental role of astrocyte–oligodendrocyte interplay in AxD and that pathological mechanisms are reversible and targetable *in vitro*. Overall, the astrocytes carrying a mutant form of GFAP are causative of increased oligodendrocyte death and reduced transition of OPCs into myelinating oligodendrocytes.

### Astrocyte–Microglia Crosstalk in Alexander Disease

There is substantial evidence for both astrocyte and microglial activation in AxD. Immunostaining with Iba1 and Ki67 monoclonal antibodies revealed enlarged and more numerous microglia in both the hippocampus and spinal cord of the GFAP^Tg^; GFAP^±R236H^ mouse model, partly due to increased proliferation rates. In the same study, Western blot confirmed that Iba1 protein levels were indeed increased in AxD mice hippocampi and spinal cord, compared to wild-type littermates. Furthermore, the observed microglial activation colocalized with GFAP immunoreactivity and astrocyte enlargement, pointing at astrocyte-microglia crosstalk in AxD (Hagemann et al., 2005). As described in *Neuropathology of Alexander Disease*, it became clear that stressed astrocytes are causative of microglia activation in AxD. A number of RNA transcripts, related to inflammatory processes, were elevated in both astrocytes and microglia. Transcripts, however, do not always accurately relate to the protein levels. ELISA arrays confirmed the increased expression of proinflammatory mediators CXCL10, CCL2, and CXCL1 at the protein level. CXCL10 upregulation was exclusively attributed to AxD astrocytes and followed GFAP immunoreactivity. CCL2 was observed in both AxD astrocytes and AxD microglia. On the contrary, the CX3CR1 and CD200R receptors were increased, which contribute to anti-inflammatory signaling. However, the expression of their respective (anti-inflammatory) ligands, CX3CL1 and CD200, was decreased in AxD mice (Olabarria et al., 2015), resulting in a reduced inhibition of microglia activation. Thus, studies in mice harboring a *GFAP* gain-of-function clearly pointed out activation of both astrocytes and microglia as a part of AxD pathology (Hagemann et al., 2005; Olabarria et al., 2015), which is in agreement with the observations from patient brain tissue (Sosunov et al., 2018). However, little attention has been paid to the functional consequences of pathological crosstalk between astrocytes and microglia in the pathophysiology of AxD. By means of CCL2 and CXCL10 expression, activated astrocytes recruit microglia to sites of injury (Tanuma et al., 2006) and facilitate the entry of T-lymphocytes into the central nervous system (CNS) (Fife et al., 2001; Klein et al., 2005; Semple et al., 2010). Consistently, in AxD, the accumulation of lymphocytes in proximity to blood vessels was described (Olabarria et al., 2015). Arrived at the target site, activated microglia can exert a dual function, ranging from neuroprotective to neurotoxic (Li et al., 2007; Gomes-Leal, 2012). AxD microglia positive for Iba1 colocalized with GFAP, indicating phagocytosis of reactive astrocytes by microglia (Sosunov et al., 2018). It is reasonable to argue that microglia in AxD, due to sustained stimulation, present themselves mainly with a detrimental phenotype. However, what is not fully consistent with this is the observation that various pro- and anti-inflammatory mediators, such as TNFα, IL-1β, IL-6, interferon-gamma (IFNγ), and IL-4 and IL-10, respectively, do not show differential expression compared to control (Olabarria et al., 2015). Important to note, data on inflammatory mediators in human AxD astrocytes and microglia are still elusive and might be an interesting avenue for future research to further understand the pathophysiology of this disease. In addition to cytokines and chemokines, expression of damage-associated molecular patterns (DAMPs), including hsp27, also induce microglial activation (Singhal et al., 2014), complicating their crosstalk even more. To conclude and to highlight the importance of future research in this direction: the functional consequence of astrocyte and microglia activation in AxD could well relate to the clinical signs observed in patients, such as their susceptibility to seizures, and is therefore an important path for future investigations.

### Microglia–Oligodendrocyte Crosstalk in Alexander Disease

Activated microglia may be protective and harmful toward myelin-producing cells. In *Microglia–Oligodendrocyte Crosstalk in Vanishing White Matter*, it is briefly discussed how activated microglia influence oligodendrocyte physiology. In contrast to VWM, activated microglia play a substantial role in the pathophysiology of AxD. Sustained activation of microglia might lead to oligodendrocyte damage. Due to their high production rate of myelin, oligodendrocytes are highly vulnerable to oxidative stress. On the other hand, (stressed) oligodendrocytes can modify the degree of microglial activation by secretion of cytokines, chemokines, and chaperokines (Peferoen et al., 2014). As an example, CXCL10 is not only secreted by astrocytes (Tanuma et al., 2006) but also oligodendrocytes (Balabanov et al., 2007) and recruits microglia to sites of injury. To date, no studies are available that specifically investigated microglia–oligodendrocyte crosstalk in AxD disease models. As astrocyte–microglia–oligodendrocyte communication is highly intertwined, *in vitro* models eliminating the effects of neurons and astrocytes might be a promising strategy to investigate AxD microglia–oligodendrocyte crosstalk solely.

## Neuropathology of Megalencephalic Leukoencephalopathy with Subcortical Cysts and CLC-2 Related Disease

The spectrum of astrocytopathies also includes MLC (van der Knaap et al., 1996). MLC pathology is usually the result of autosomal recessive inherited mutations in the *MLC1* or *GLIALCAM* (MLC2A) gene (Topcu et al., 2000), of which new variants have increasingly been identified in the past years (Leegwater et al., 2001, 2002; Ben-Zeev et al., 2002; Kariminejad et al., 2015; Abdel-Salam et al., 2016; Cao et al., 2016; Shi et al., 2019). The clinical course of the classic form of MLC is characterized by development of macrocephaly during the first years of life, progressive motor deterioration, ataxia, cognitive decline, and epilepsy. The second known form of MLC is one with a remitting phenotype of macrocephaly and associated with dominant mutations in the *GLIALCAM* gene. However, years after the age of onset, patients experience progression of ataxia, spasticity, and cognitive decline and are susceptible to seizures (van der Knaap and Bugiani, 2017). Imaging findings typically involve diffusely abnormal, swollen white matter, as illustrated by diffusion restriction on MRI, and the presence of cysts, with a preferred location in the temporal horns and frontoparietal regions. Histopathological examination showed numerous vacuoles in the cerebral white matter, and the use of electron microscopy confirmed the disintegration of myelin layers and intramyelinic formation of vacuoles. Immunostaining of MBP revealed no abnormalities in the amount of myelin. Although fibrillary astrogliosis was detected, microglial activation was relatively absent. In areas of gliosis, MLC1 is more abundantly expressed around the capillaries. Astrocytosis was only found in the molecular layer of the cortex, which is most likely a result of experienced epileptogenic insults (van der Knaap et al., 1996; Boor et al., 2005; van der Knaap and Bugiani, 2017). Only one brain autopsy of an MLC patient has been performed and published to date. This patient carried a homozygous c.206^C>T^ mutation. Tissue examination was in line with the above described characteristics for MLC. Mutated MLC1 was detected in intracellular compartments, instead of at the glial cellular membrane. A reduction in the number of astrocytes and oligodendrocytes and a mild infiltration of leukocytes were observed. Furthermore, accumulations of αB-crystallin, but not RFs (López-Hernández et al., 2011), were seen in the cytoplasm of glia. The presence of αB-crystallin is indicative of cellular stress (Head et al., 1994). CLC-2-related disease is similar to MLC in terms of pathophysiology, although it is caused by either homozygous or heterozygous mutations in the *CLCN2* gene. Like MLC1, CLC-2 chloride channels are mainly located in the astrocytic end-feet enwrapping blood vessels. CLC2-related disease shows similarities with MLC, albeit patients harboring mutations in *CLCN2* genes do not experience epilepsy (Depienne et al., 2013). In *Astrocyte–Oligodendrocyte–Microglia Crosstalk in Megalencephalic Leukoencephalopathy With Subcortical Cysts*, the consequences of astrocytic dysfunction in MLC and CLC-2-related disease will be collectively discussed.

### Astrocyte–Oligodendrocyte–Microglia Crosstalk in Megalencephalic Leukoencephalopathy With Subcortical Cysts

Megalencephalic leukoencephalopathy with subcortical cysts 1 is a membrane protein that is exclusively expressed in the distal processes of perivascularly located astrocytes and in subependymal and subpial regions and in Bergmann glia of the cerebellum (Boor et al., 2005). GlialCAM is known as its chaperone protein and necessary for accurate localization of MLC1 (López-Hernández et al., 2011; Capdevila-Nortes et al., 2013). The localization of MLC1, together with the observation of increased water content in the MLC1- or GlialCAM-deficient brain, hinted toward a role in the regulation of ion–water homeostasis. Research into the function of GlialCAM and MLC1 showed that these proteins are responsible for adequate regulation of fluid excesses after neuronal depolarization. To this end, MLC1 interacts with other molecules, such as the β subunit of the Na^+^/K^+^-ATPase pump, Kir4.1, AQP4, syntrophin, dystrobrevin, caveolin-1, and TRPV4. For a detailed discussion on the function of GlialCAM and MLC1, and the molecules they interact with, the reader is referred to a review by Brignone et al. (2015) (Brignone et al., 2015). It was hypothesized that a lack of functional GlialCAM/MLC1 interferes the transportation of ions across the cellular membrane, what leads to disrupted cellular volume regulation and presents with a swollen astrocyte phenotype. As MLC1 is expressed in astrocyte–astrocyte contacts and not oligodendrocytes, the myelin structure damage observed in MLC disease is the result of a primary astrocyte defect (Schmitt et al., 2003; Boor et al., 2005; Teijido et al., 2007). Yet, the consequences for molecular crosstalk of MLC1- or GlialCAM-deficient astrocytes with oligodendrocytes and microglia are not clear. Thus, the following hypotheses on interglial communication in MLC- and CLC2-related disease are based on indirect evidence. First, it is thought that MLC1 has a close relation with the formation and stability of myelin, as it is highly conserved in vertebrates that possess a myelinated brain (Boor et al., 2005) and most abundantly expressed during active myelination (Dubey et al., 2015). Furthermore, astrocyte-specific MLC1 expression is responsible for correct localization of GlialCAM in oligodendrocytes (Hoegg-Beiler et al., 2014). Impaired functionality of GlialCAM in oligodendrocytes might affect the physiology of oligodendrocytes in such a way that it is contributing to the pathophysiology of MLC. In addition, albeit Kir4.1 expression is not altered in astrocytes obtained from a MLC1 knockout mouse model (Bugiani et al., 2017), it might be interesting to investigate the oligodendrocyte Kir4.1 expression in a coculture design with MLC1-deficient astrocytes, as this ion channel was found to be involved in oligodendrocyte buffering of potassium and controlling the seizure susceptibility threshold but did not alter the myelin structure, which is representative of MLC pathology. A second interesting angle to approach the possibly impaired interglial crosstalk in MLC is by assessment of glial gap junctions. Astrocyte–astrocyte and astrocyte–oligodendrocyte communication is facilitated by gap junctions build of connexin proteins (Nutma et al., 2020). Interestingly, it was found that GlialCAM is required for Cx43 localization to the astrocyte plasma membrane (Wu et al., 2016). Lutz et al. (2009) investigated the effect of astrocyte-specific knockout of Cx43 and Cx30 on oligodendrocyte maturity and myelin production. Double knockout mice showed edematous astrocytes, vacuolated oligodendrocytes, and edema within myelin sheaths. This phenotype had an onset at postnatal day 23 and persisted into adulthood. In addition, the lack of Cx43 and Cx30 in astrocytes was associated with impaired myelination, as illustrated by reduced MBP expression. Thus, it was proposed that a loss of astrocyte-specific gap junctions can cause oligodendrocyte and myelin pathology (Lutz et al., 2009). The true effect of astrocyte connexins on oligodendrocytes was examined by Magnotti et al. (2011) using mice having a mutation in both an oligodendrocyte connexin (Cx47 or Cx32) and an astrocyte connexin (Cx43). Cx43/Cx32^–/–^ mice presented with a phenotype of myelin vacuolation (Magnotti et al., 2011). In addition, a significant loss of astrocytes was observed in these mice compared to wild-type controls. Oligodendrocyte counts, however, were not changed. The observed pathology was related to the presence of seizures and an increased premature mortality rate. Together, these studies highlight the possibility that disconnection of astrocyte–astrocyte and astrocyte–oligodendrocyte contacts is causative of edema formation and myelin sheath damage in MLC. Finally, it is interesting but unclear how astrocyte activation is present in MLC pathology but does not induce microglia activation. Recently, mutated *MLC1* was associated with altered cellular morphology and motility due to modifications of cytoskeletal proteins (Hwang et al., 2019). While modification of the astrocyte cytoskeleton in AxD led to an inflammatory response and promoted microglia activation, MLC tissue specimens do not show activated microglia. As described earlier, it is believed that astrocyte reactivity precedes microglia activation; therefore, it might be interesting to map the inflammatory secretome of MLC1- or GlialCAM-deficient astrocytes and investigate the effect of the secreted molecules on microglia.

In summary, the currently available research underscores the necessity of MLC1, GlialCAM, and CLC-2 in adequate ion–water homeostasis. Lack of function of these proteins severely interferes with the capability of astrocytes to encounter hyposmotic conditions and could secondarily affect glial cell crosstalk. Insights in the consequences of MLC1 or GlialCAM mutations on the capability of astrocytes to support oligodendrocytes and to activate microglia are very limited. Advanced *in vitro* model systems using MLC1-, GlialCAM-, or CLC-2 deficient astrocytes cocultured with other cells of glial lineage (e.g., primary cultured oligodendrocytes or microglia from MLC patients or iPSC generated oligodendrocytes) could deepen our understanding of the pathophysiological mechanisms of MLC- and CLC-2-related disease.

## Neuropathology of Aicardi–GoutièRes Syndrome

Aicardi–Goutières syndrome is a rare familial, but genetically heterogeneous, early onset encephalopathy with a severe disease course. The condition was first described by Aicardi and Goutieres (1984). Clinically, AGS patients present with vomiting and feeding difficulties, spasticity, dystonia, abnormal eye movements, progressive neurological deterioration, and acquired microcephaly. Non-neurological signs include hepatosplenomegaly and necrotic skin lesions (known as “chilblains”) (Goutières, 2005). Furthermore, chronic lymphocytosis and elevated levels of the cytokine interferon-alpha (IFNα) have been detected in the cerebrospinal fluid of patients. On imaging modalities, AGS patients exhibit intracranial calcifications in the basal ganglia, dentate nuclei of the cerebellum, and periventricular white matter. These calcifications can be detected from the first day of life; however, cortical atrophy and deep white matter hypodensities, which are also features of this disease, appear later (Goutières, 2005). Histopathological examinations of AGS brain tissue are very limited (Barth et al., 1999), but a sequence of six diagnostic criteria have been determined, including (Bellot-Saez et al., 2017) severe microcephaly; (Wang and Bordey, 2008) diffuse lack of myelin with highly variable intensity; (Dietschy and Turley, 2004) calcific depositions in the white matter, thalami, basal ganglia, and dentate nuclei; (Howarth, 2014) calcification in the walls of small vessels; (van der Knaap and Bugiani, 2017) cortical microinfarctions; and (Bugiani et al., 2010) tissue inflammation in necrotic areas and leptomeninges (Barth, 2002). Regarding genetics, homozygous mutations in *TREX1*, *RNASEH2A*, and *RNASEH2B* were initially associated with the development of AGS (Rice et al., 2007). Four additional genes have been added to the list of AGS causative genes, including *RNASEH2C*, *SAMHD1*, *ADAR1*, and *IFIH1/MDA5* (Livingston et al., 2013). Sase et al. (2018) overviewed the cellular pathways leading to the AGS phenotype and concluded that the underlying pathophysiological mechanism concerns increased production and dysregulated control of IFNα levels by activation of the cyclic GMP–AMP synthase (cGAS)/stimulator of interferon genes (STING) pathway (Sase et al., 2018). As astrocytes are a major source of IFNα production, this condition is now considered an astrocytopathy (van der Knaap and Bugiani, 2017). Indeed, brain tissue examination of AGS patients demonstrated colocalization of GFAP with cytokines IFNα and CXCL10 (van Heteren et al., 2008). Although the effects of AGS-causing gene mutations on astrocyte physiology and astrocyte–oligodendrocyte as well as astrocyte–microglia crosstalk are still an open question; in *Astrocyte–Oligodendrocyte Crosstalk in Aicardi–Goutières Syndrome* and *Astrocyte–Microglia Crosstalk in Aicardi–Goutières Syndrome*, respectively, possible mechanisms of action are discussed.

### Astrocyte–Oligodendrocyte Crosstalk in Aicardi–Goutières Syndrome

Astrocytes harboring an AGS-associated mutation might influence oligodendrocyte function in a number of ways. First, AGS astrocytes are relatively more vulnerable to cell death (Sase et al., 2018). As a consequence, lower numbers of astrocytes are less capable of providing sufficient trophic support to oligodendrocytes, thereby negatively influencing the myelinating capacity and survival of oligodendrocytes. Indeed, neuropathological examination of a patient carrying a SAMHD mutation demonstrated increased numbers of caspase-3-positive oligodendrocytes, mildly reduced counts of olig-2-positive oligodendrocytes, and marked lack of myelin in the white matter. In contrast, the number of OPCs (PDGFRα+) was increased in AGS specimens compared to the tissue of a healthy control (Klok et al., 2015). Second, astrocytes harboring an AGS-causing mutation show substantial astrogliosis, release more of cytokines IFNα and CXCL10, and induce the expression of interferon-stimulated genes (Sase et al., 2018). Although it is known that type II IFNs (e.g., IFNγ) are detrimental to oligodendrocytes (Popko and Baerwald, 1999), little is known about the possible impact of type I IFNs (e.g., IFNα) on oligodendrocytes. Third, it was shown that long-term treatment of human neural stem-cell-derived astrocytes with IFNα led to reduced proliferation rates and promotion of activation. In addition, it was reported that IFNα treatment *in vitro* for 21 days resulted in a reduction in *ATF4, EIF2B1*, and *CTSD* mRNA expression (Cuadrado et al., 2013). The *CTSD* gene codes for an enzyme cathepsin D, which transports PLP to the cell membrane. Recently, it was shown that cathepsin D knockout mice demonstrate delayed differentiation of OPCs into mature oligodendrocytes, a reduced number of mature oligodendrocytes (CC1+), and that the oligodendrocytes have difficulties with trafficking PLP to the cell membrane (Guo et al., 2018). Furthermore, *ATF4* and *EIF2B1* loss of function are also involved in the pathogenesis of VWM and associated with a severe lack of mature oligodendrocytes and astrocytes (Bugiani et al., 2010). In conclusion, the currently established literature points out that astrocyte-mediated production of cytokines and altered gene expression in AGS is able to negatively affect oligodendrocyte maturation, survival, and myelinating capacity. To confirm this theory and to gain additional insights in glial crosstalk in AGS, a direction for future research could be to expose astrocytes to double-strand RNA (dsRNA) polyinosinic:polycytidylic acid (poly I:C) (this is expected to upregulate their production of IFNα) and subsequently assess the consequences for oligodendrocyte functionality in a coculture setup. Finally, a direct effect of AGS mutations to oligodendrocyte physiology has neither been confirmed nor ruled out. Therefore, it might be interesting to test the effect of, for example, knock-in mutations in *TREX1* or other AGS-related genes, on the maturation of OPCs or the myelinating capacity of mature oligodendrocytes to include or exclude oligodendrocyte-autonomous mechanisms in the neuropathology of AGS.

### Astrocyte–Microglia Crosstalk in Aicardi–Goutières Syndrome

Reactive microgliosis was observed in areas of microinfarction in the AGS brain (Barth et al., 1999). Sase et al. (2018) proposed that microglia in the AGS brain might be activated by astrocyte-mediated production of type I IFNs or in a cell autonomous fashion (Sase et al., 2018). In agreement, a review by West et al. (2019) explained the strong response of microglia in the context of sustained and elevated levels of IFNα (West et al., 2019). This strong response is characterized by a hypertrophied phenotype (West et al., 2019) and increased expression of several IFN-stimulated genes and the nuclear transcription factor IRF1 as well as a cell-surface molecule CD68 (a T-lymphocyte attractor) (Li W. et al., 2018). Although not markedly present, in the autopsy case described by Klok et al. (2015), CD68 + microglia and CD3 + lymphocytes were noted (Klok et al., 2015). In addition, it was found that the release of CXCL10 by AGS astrocytes was substantially increased compared to control (Sase et al., 2018). As previously mentioned, CXCL10 is a recruitment molecule for microglia to sites of injury (Tanuma et al., 2006). Albeit the recruited microglia are able to phagocyte myelin, no myelin-laden macrophages were found in the AGS brain (Klok et al., 2015). Together, microglia in AGS contribute to a dysregulated immune response in the CNS, thereby exerting detrimental effects on the white matter integrity. Important to note, *TREX1* expression was also found in a subset of microglia that has a close relationship with the microvasculature in the human brain (Kothari et al., 2018). It might thus be of interest to examine the effect of TREX1 loss-of-function mutations in microglia, as this might shed light on other, non-astrocyte, induced ways of microglia-mediated pathology development in AGS.

## Neuropathology of Oculodentodigital Dysplasia

Oculodentodigital dysplasia is a rare genetic condition that manifests with anomalies to the eyes (oculo-), teeth (dento-), and fingers (digital-) (Gorlin et al., 1963; Cavusoglu et al., 2019) as well as neurological features (Loddenkemper et al., 2002). Neurological symptomatology is present in approximately 30% of the patients and includes dysarthria, lack of bladder control, spastic para- or tetraparesis, ataxia, muscle weaknesses, seizures, and mental retardation. Homozygous mutations in the *GJA1* gene locus have been causally related to the disease onset (Paznekas et al., 2003; Huang et al., 2013). The *GJA1* gene encodes the gap junctional hemichannel Cx43, which is present multiple cell types (Laird, 2006), among which astrocytes (Dermietzel and Spray, 1993). Therefore, ODDD is regarded as an astrocytopathy (van der Knaap and Bugiani, 2017). Recently, it was discovered that Cx43 regulates the neural crest cell epithelial-to-mesenchymal transition (Jourdeuil and Taneyhill, 2020), which explains the widespread symptomatology in ODDD patients. Neurological signs result from altered Cx43 hemichannel expression on the astrocytic cell membrane. Important to note, different ODDD-linked mutations affect different domains of the Cx43 molecule, therefore having a variable effect on Cx channel properties and result in a variety of neurological symptoms (De Bock et al., 2013). In general, Cx43–Cx43 as well as Cx43–Cx30 gap junctions were found to be necessary for astrocyte–astrocyte communication, and, among others, Cx43–Cx47 gap junctions mediate astrocyte–oligodendrocyte communication (Nagy and Rash, 2000). A wide variety of functions were attributed to these heterotypic and homotypic gap junctions, including the propagation of calcium (Ca^2+^) waves across the astrocytic network, buffering of K^+^ and maintenance of cellular glucose levels (De Bock et al., 2013).

*In vivo* and *in vitro* models shed light on the consequences of mutated or absent Cx43. In 2003, a study by Theis et al. (2003) showed that astrocyte-specific deletion of Cx43 negatively influenced astrocyte–astrocyte interactions, but it did not completely abolish the communication. In addition, no alterations in astrocyte viability, morphology and reactivity were observed upon depletion of Cx43 (Theis et al., 2003). Behavioral consequences of Cx43 knockout in mice included gait and motor disturbances, similar to ODDD (Frisch et al., 2003). Introduction of ODDD-specific Cx43 mutations in glioma cells confirmed an altered Cx43 hemichannel structure and dysfunctional gap junctions (Lai et al., 2006). A description of the effects of ODDD-linked mutations on a cellular level in humans is still elusive, as there are no ODDD brain autopsies or biopsy reports available.

Based on the currently available literature, altered Cx43 expression on astrocytes might impact astroglial crosstalk on several levels, for example by unbalanced K^+^ and Ca^2+^ concentrations (Fannon et al., 2015; Gautier et al., 2015) and changes in the immune response (Morioka et al., 2018; Vignal et al., 2020). In *Astrocyte–Oligodendrocyte Crosstalk in Oculodentodigital Dysplasia* and *Astrocyte–Microglia Crosstalk in Oculodentodigital Dysplasia*, plausible effects of mutated Cx43 on oligodendrocytes and microglia are discussed and put in relation to ODDD pathophysiology.

### Astrocyte–Oligodendrocyte Crosstalk in Oculodentodigital Dysplasia

Oligodendrocytes are highly dependent on astrocytic trophic support, which is mediated by heterotopic gap junctional connections (Cx43/Cx47 and Cx30/Cx32). The Cx43/Cx47 combination is the most present gap junctional combination in the white matter (Orthmann-Murphy et al., 2008) and regulates K^+^ and glutamate excesses after neuronal depolarization, is involved in lactate distribution, and propagates of Ca^2+^ waves across the glial cell network (De Bock et al., 2013; Basu and Sarma, 2018). Thus, Cx43 loss of function might affect oligodendrocyte function in a number of ways. For example, as oligodendrocytes are very sensitive to K^+^ and Ca^2+^ levels, alterations in the ionic and nutrient homeostasis may have detrimental effects on their physiology and survival. In addition, the myelinating capacity of oligodendrocytes can be affected by astrocytic depletion of Cx43. May et al. (2013) showed that oligodendrocyte expression of Cx47 is dependent on Cx43 expression by astrocytes. The data suggested that Cx43 expression regulates posttranslational phosphorylation of Cx47, which is an essential step in its functionality (May et al., 2013). Functional Cx47 is critical for the process of myelination by oligodendrocytes (Basu and Sarma, 2018). Thus, Cx43 loss of function in ODDD might affect oligodendrocyte survival and their myelinating capacity. An astrocyte–oligodendrocyte *in vitro* model similar to the one that was used by Li L. et al. (2018) (described in *Astrocyte–Oligodendrocyte Crosstalk in Alexander Disease*) might provide new insights in ODDD pathophysiology (Li L. et al., 2018).

### Astrocyte–Microglia Crosstalk in Oculodentodigital Dysplasia

Recent research pointed out a modulating role for astrocyte-specific Cx43 in the CNS immune response (Boulay et al., 2015, 2016). Vignal et al. (2020) investigated the impact of astrocyte-specific Cx43 knockout on the emergence of a neuroinflammatory response. Cx43-deficient mice and control mice (harboring functional Cx43) were treated with LPS and examined on the translocator protein (TSPO) using positron emission tomography (PET), which is considered a tool to assess microglial activation (Guilarte, 2019; Vignal et al., 2020). Systemic injection of LPS resulted in an increased PET-TSPO signal in control mice but not in Cx43-depleted mice. Consistently, western blot assessment of TSPO protein expression after LPS stimulation indicated an elevation of TSPO in control mice but not in Cx43 knockout mice. The authors concluded that astrocyte-specific knockout of Cx43 prevented an LPS-induced neuroinflammatory response (Vignal et al., 2020). At present, it is unknown to what extent *GJA1* mutations affect the astrocyte- and microglia-mediated inflammatory response in the human brain. A suggestion for future research might be to introduce ODDD-associated mutations in cultured human astrocytes and to subsequently assess their potency to activate microglia. Another interesting aspect is the influence of Cx43 depletion from astrocytes on the blood–brain barrier (BBB) endothelial cells. It was found that the absence of Cx43 from astroglia prompts peripheral immune cells to migrate across the BBB (Boulay et al., 2015). The authors of this study investigated whether the immune cell infiltration was due to a reduced BBB integrity or astrogliosis, but no indications were found for either of them. On the other hand, increased expression of endothelial activation markers (e.g., ICAM-1, VCAM-1, and P-Selectin) were detected by qPCR using blood vessel samples of Cx43 knockout mice. In addition, elevated chemokine expression (CCL5, CXCL10, and CXCL12) was observed in the CNS tissue of Cx43 knockout mice compared to control. Therefore, it was suggested that the absence of Cx43 in astrocytes lead to endothelial activation and chemoattraction of leukocytes into the brain tissue. Interestingly, the entry of peripheral leukocytes into the CNS induced an autoimmune response against Vwa5a, an extracellular matrix protein produced by astrocytes; however, no evident tissue breakdown was observed (Boulay et al., 2015). Follow-up research from the same group revealed that depletion of Cx43 results in a differential astrocyte-specific transcriptome (Boulay et al., 2018), which does not match the classic A1 and A2 reactivity states (Anderson et al., 2016; Liddelow et al., 2017). The discovery of this unique astrocytic phenotype strengthens the suggestion to investigate astrocyte–microglial crosstalk in an ODDD disease model. Neuropathological reports on ODDD are needed to address the value of the presented research for ODDD patients.

## Conclusion

Astrocytes act as bridges among all kinds of cells in the CNS, including neurons, oligodendrocytes, microglia, endothelial cells, and astrocytes themselves. Interglial communication is highly complex, multidirectional, and dependent on the microenvironment. Increasing knowledge on the numerous roles of astrocytes in development, maintenance of homeostasis, and response to injury suggests that disruption of normal astrocyte functions, astrocyte degeneration, or dysfunctional maladaptive astrogliosis can be driving factors in the pathophysiology of many neurological diseases. The purpose of this review was to address the mechanisms by which a primary astrocyte defect could lead to microglia, oligodendrocyte, and myelin production pathologies, as many astrocytopathies present as a leukodystrophy phenotype. Although the astrocytopathies included in this review have a unique genetic etiology, there are shared pathophysiological mechanisms, among which are accumulations of aberrantly folded or alternatively spliced proteins, modified secretion of ECM molecules, altered secretion of pro- and anti-inflammatory molecules, cytokines and chemokines, alterations in the cellular gap junctional network, and a changed ionic and nutrient homeostasis. Interestingly, a variable spectrum of astro- and microgliosis is observed in the different astrocytopathies (Table 1). In summary, VWM is characterized by an immature, non-activated astrocytic phenotype and an increased UPR. There is evidence for an astrocyte-mediated reduction in oligodendrocyte maturation and inhibition of microglial activation. Accumulations of hyaluronan were suggested to play a leading role in this process. In contrast, in AxD, activation of astrocytes and microglia is observed, which comes along with increased levels of oxidative stress, inhibition of proteasome function, and endoplasmic reticulum stress. Impaired control of ionic homeostasis by astrocytes and their secretion of hyaluronan and CHI3L1 relates to increased rates of oligodendrocyte apoptosis and reduced OPC maturation, thus negatively affecting the myelinating capacity. The hallmark of MLC is white matter vacuolization, and although astrogliosis is observed, microgliosis is not. How these MLC-specific characteristics are relatable to each other is currently not clarified. An underlying mechanism could probably be found in a disturbed glial cell gap junctional network. AGS presents itself also with substantial astrogliosis. AGS-associated gene mutations cause astrocytes to produce elevated levels of IFNα, which in turn causes delayed OPC differentiation, oligodendrocyte apoptosis, a reduction in the myelinating capacity, and sustained activation of microglia. In ODDD, Cx43 loss of function results in aberrant ionic and nutrient homeostasis, as it detrimentally affects astrocyte–astrocyte and astrocyte–oligodendrocyte contacts via gap junctions. In addition, depletion of Cx43 was associated with a dysregulated immune response mediated by astrocytes and microglia, as well as endothelial cell activation, which might ultimately result in entry of peripheral leukocytes into the CNS tissue. Altogether, defective functioning of astrocytes results in variable levels of astrogliosis and microgliosis, and, directly or indirectly, oligodendrocyte function is affected. An improved understanding of the shared and unique pathophysiological mechanisms of astrocytopathies could contribute to the development of treatment strategies, as there are currently none available.

**TABLE 1:** Overview of commonalities and differences in the pathophysiology of the various astrocytopathies.

Astrocytopathy	Astrocytes	Oligodendrocytes	Microglia	Pathophysiological highlights	
Vanishing white matter	Reduced astrocyte count in subcortical U-fibers Immature and non-activated phenotype: broad, blunt processes Astrogliosis −	Increased OPC:mature oligodendrocyte ratio	Microgliosis −	Myelin thinning and lack of myelin, myelin vacuolation, tissue rarefaction and cavitation Elevated activation of the glial UPR Cytoskeleton defects	Defective mitochondrial function (decreased oxidative phosphorylation) Altered composition of ECM molecules (hyaluronan) Compromised inflammatory response in astrocytes and microglia Oligodendrocyte maturation defect
Alexander disease	No changes in astrocyte count Activated phenotype and presence of Rosenthal fibers in the cytosol Astrogliosis ++	OPC maturation is halted Less mature oligodendrocytes Decreased oligodendrocyte survival rate due to defects in ionic buffering in the extracellular milieu	Microgliosis ++	Lack or loss of myelin Compromised mitochondrial function Increased levels of cellular oxidative stress	Proteasome dysfunction Endoplasmic reticulum stress →Contribution to glial cell reactivity Impaired control of ionic balance in extracellular milieu Altered composition of ECM molecules (hyaluronan and CHI3L1)
Megalencephalic leukoencephalopathy with subcortical cysts	Astrocyte count is reduced Swollen phenotype due to disrupted cellular volume regulation Astrogliosis +	Oligodendrocyte count is reduced	Microgliosis −	Vacuolization of the cerebral white matter. The amount of myelin is not changed Dysregulation of ion-water homeostasis	Altered glial gap junctional network
Aicardi–Goutières syndrome	Reduced astrocyte count Astrogliosis +	Increased OPC:mature oligodendrocyte ratio Increased oligodendrocyte apoptosis rate	Microgliosis ±	Lack of myelin Increased production and dysregulated control of IFNα levels Intracranial calcifications as well as calcification in the walls of small vessels, severe microcephaly, lack of myelin, cortical microinfarctions, tissue inflammation	
Oculodentodigital dysplasia	*Unknown*	*Unknown*	*Unknown*	Reduced expression of gap junctional hemichannel Cx43, with presumably alterations in the glial gap junctional network as a consequence A presumed dysregulated immune response mediated by astrocytes and microglia	

*Per astrocytopathy observations regarding astrocytes (count, phenotype, and extent of reactivity), oligodendrocytes (effect on maturation and survival), and microglia (extent of reactivity) are summarized. The presence or absence of astrogliosis and microgliosis is indicated by −, ±, +, or ++. Furthermore, important pathophysiological mechanisms are enumerated in the right column. OPC, oligodendrocyte precursor cells; UPR, unfolded protein response; IFNα, interferon α; ECM, extracellular matrix molecules.*

## Author Contributions

DdW performed the literature search and review, and wrote the first draft of the manuscript. DdW and MB edited and contributed to all subsequent drafts. Both authors approved the submitted version.

## Conflict of Interest

The authors declare that the research was conducted in the absence of any commercial or financial relationships that could be construed as a potential conflict of interest.
